# Parametric Analysis of the Mandrel Geometrical Data in a Cold Expansion Process of Small Holes Drilled in Thick Plates

**DOI:** 10.3390/ma12244105

**Published:** 2019-12-08

**Authors:** Jose Calaf-Chica, Marta María Marín, Eva María Rubio, Roberto Teti, Tiziana Segreto

**Affiliations:** 1 Department of Civil Engineering, University of Burgos, Av. Cantabria s/n, 09007 Burgos, Spain; 2 Department of Manufacturing Engineering, Universidad Nacional de Educación a Distancia (UNED), St. Juan del Rosal 12, E28040 Madrid, Spain; mmarin@ind.uned.es (M.M.M.); erubio@ind.uned.es (E.M.R.); 3 Department of Chemical, Materials and Industrial Production Engineering, University of Naples Federico II, Piazzale Tecchio, 80, 80125 Naples, Italy; roberto.teti@unina.it (R.T.); tsegreto@unina.it (T.S.)

**Keywords:** cold expansion, mandrel, cold-forming, swaging

## Abstract

Cold expansion technology is a cold-forming process widely used in aeronautics to extend the fatigue life of riveted and bolted holes. During this process, an oversized mandrel is pushed through the hole in order to yield it and generate compressive residual stresses contributing to the fatigue life extension of the hole. In this paper, a parametric analysis of the mandrel geometrical data (inlet angle straight zone length and diametric interference) and their influence on the residual stresses was carried out using a finite element method (FEM). The obtained results were compared with the conclusions presented in a previous parametric FEM analysis on the influence of the swage geometry in a swaging cold-forming process of gun barrels. This process could be considered, in a simplified way, as a scale-up of the cold expansion process of small holes, and this investigation demonstrated the influence of the diameter ratio (K) on the relation between the mandrel or swage geometry and the residual stresses obtained after the cold-forming process.

## 1. Introduction

The autofrettage cold-forming process for thick-walled cylinders has its origin in artillery technology of the 19th century. Gun barrels showed a significant development in range, power and reliability because of the application of an innovative manufacturing process: the generation of internal compressions at the bore with a hoop installed on the barrel with enough interference. In the 1920s, a new cold-forming process was developed to obtain similar residual compressive stresses using a hydraulic pressure into the barrel. It was called hydraulic “autofrettage” using a French term which means “self-hooping” due to the absence of hoops to obtain the residual stresses [1]. Development of new steel alloys during the first half of the 20th century with higher yield and ultimate tensile strengths generated an increment of the necessary autofrettage pressure to reach the requirements of residual stresses. The inherent process difficulties to reach these pressure levels motivated the development of alternative processes (explosive autofrettage [2,3], double-layer autofrettage [4], thermal autofrettage [5], rotational autofrettage [6], etc.). In this context, around the middle of the last century, an innovative cold-forming process in gun barrels was presented [7]: the mechanical autofrettage or swaging. In this process, a swage with a diametrical interference with the barrel is forced to pass through bore, generating compressive residual stresses. The necessary pressure to move the swage was significantly lower than the equivalent hydraulic pressure in a hydraulic autofrettage.

The cold-forming process of swages showed alternative applications in aeronautics in the 1970s to extend the fatigue life of fastened parts on aircrafts. In 1974, Boeing’s materials research and development developed a swaging process called “coldworking” which was applied to the fastened holes to extend their fatigue life [8,9]. An oversized swage or mandrel was pushed through the hole to yield the inner diameter of the hole. The remaining elastic zone after the swaging process generated a residual compressive hoop stress which was the origin of the delay in the crack initiation. Although there are many investigations related to the effect of the cold expansion on holes [10,11] and failure mode analyses of the riveted assemblies [12], there are less studies centred on the geometrical parameters of the cold expansion process. An optimization of the mandrel geometry would derive on an increase of the compressive residual stresses obtained after the cold forming process and an increase in the fatigue life of the fastened assemblies. The cost of maintenance in the aeronautical industry is directly related to the frequency of the periodic inspections. This frequency is calculated with analytical and numerical analyses of crack propagation (fatigue and damage tolerance analyses). Most of the crack initiation locations in aeronautics are considered in fastened holes, and an aircraft is plenty of holes. The cold expansion process is a technique to expand the fatigue life of holes, retarding the initiation of a crack and slowing down the crack propagation. Therefore, this extension in fatigue life would reduce the necessary frequency of some periodic inspections, reducing the cost of maintenance, and expanding the economic life of the airplanes.

The main difference between both applications of this cold-forming process, swaging of thick-walled cylinders and cold expansion of fastened holes in aircrafts, would seem to be only in the size of the hole: big holes in thick-walled cylinders and small holes in aeronautics. Swage geometry and how it affects to the residual stresses could be similar in both applications, because it is a scale-up of a similar cold-forming process.

**Figure 1:**
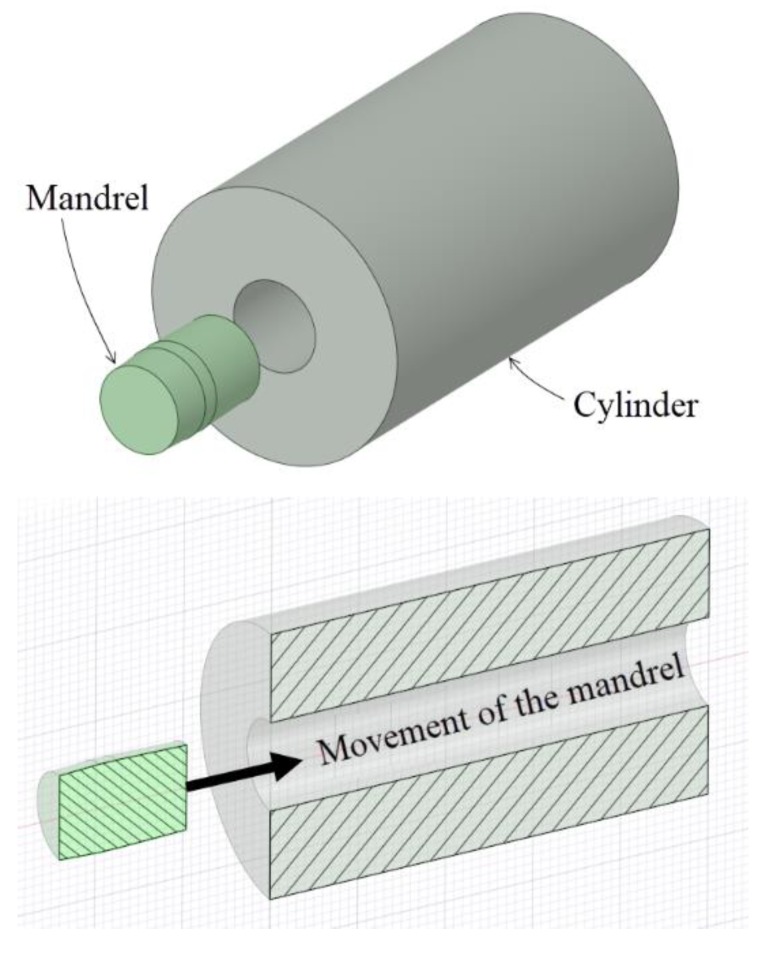
Schematic representation of the swaging process.

Figure 1 shows a schematic representation of the most important parts in a cold-forming process of swaging:

1. The swage or mandrel. A part manufactured with tungsten carbide alloy, high-strength steel or a combination of different materials, and it is pushed through the hole during the cold expansion process.
2. The cylinder or hole. The part that is cold-formed. It could be a cylinder or a hole.

**Figure 2:**
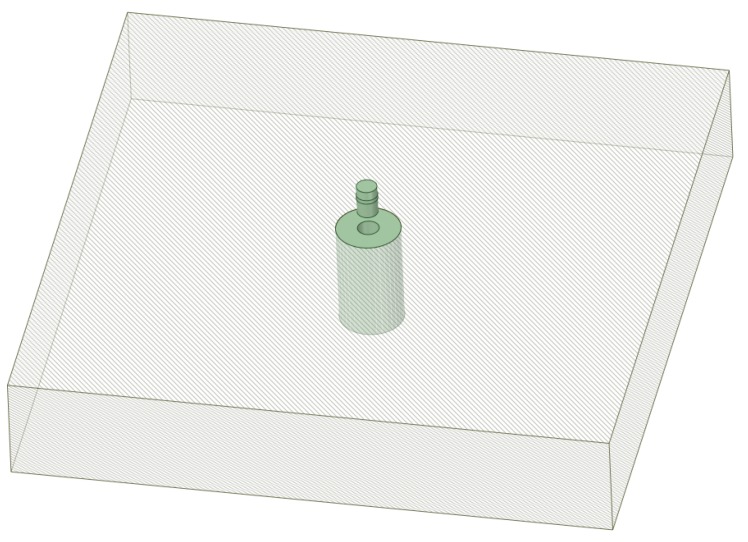
Sketch of a cold expansion process in a small hole.

Figure 2 shows the application of the swaging process in a hole drilled in a thick plate representing a sketch of the application of the cold expansion process for fastened holes in aeronautics. When the ratio between the hole diameter and the thickness of the plate is high enough (thick plates and plane strain condition), the main difference between thick-walled cylinders and holes in plates is in the *K* ratio or diameter ratio of the cylinder (*K* = *D_ext_/D_int_*). In the thick-walled cylinders, *K* is limited to small values, and it takes very high values for holes in plates.

**Figure 3:**
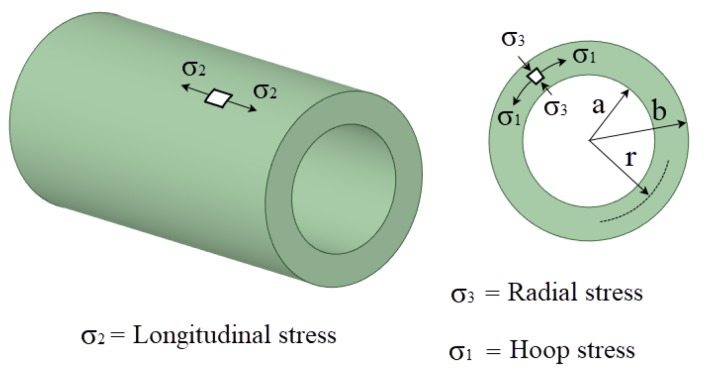
Sketch of stress field nomenclature.

**Figure 4:**
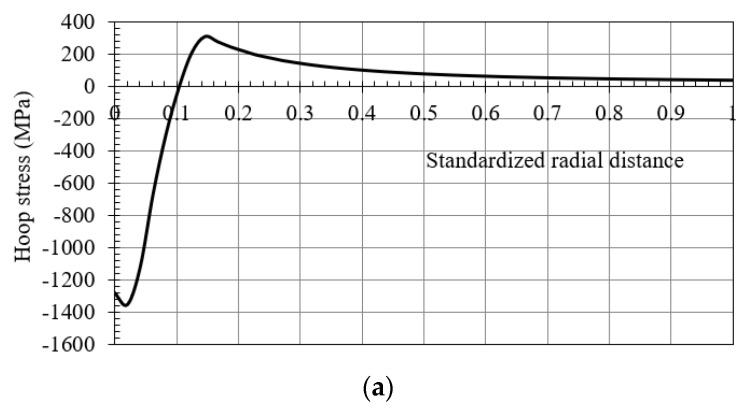

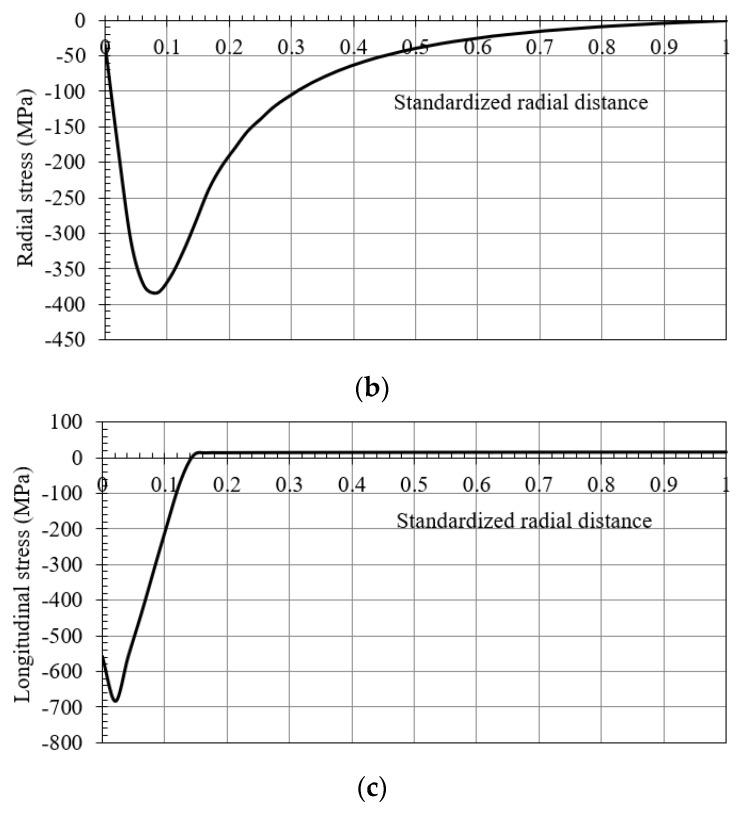
Residual (**a**) hoop, (**b**) radial and (**c**) longitudinal stresses after a cold expansion forming process.

In both cases (Figure 1; Figure 2), the residual stress field obtained after the cold-forming swaging process is typically represented in a polar coordinate system. The principal stresses (σ_1_ = hoop, σ_2_ = longitudinal, and σ_3_ = radial) are shown in Figure 3. In Figure 4 an example of the residual stress field is reported along the normalized radial distance *r* = (*D* − *Dint*)*/*(*Dext* − *Dint*) at the middle longitudinal distance of a hole. After the cold expansion forming process, a compressive residual hoop stress (Figure 4a) is generated in the inner radius of the hole preventing and retarding any crack initiation.

**Figure 5:**
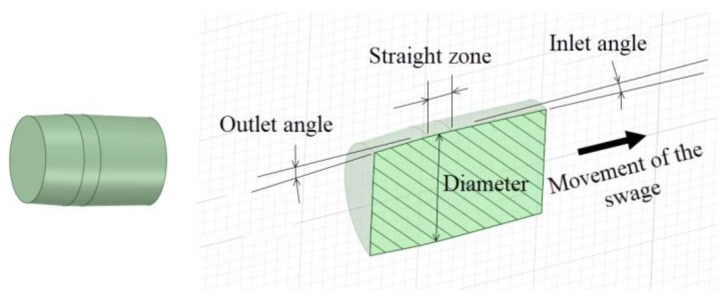
Sketch of a mandrel or swage with the geometrical parameters.

The main geometrical parameters of the mandrel are: the inlet angle (*α*), the outlet angle (*β*), the length of the straight zone (*SZ*) and the major diameter (*d_s_*) (see Figure 5). The swage major diameter is important just related to the hole diameter and the interference between them (*int*).

The most important reference for the investigation of the influence of the swage geometrical data for thick-walled cylinders was the research published by Gibson et al. [13,14]. A systematic finite element method (FEM) analysis of a swaging process in a cylinder with *K* = 2.257 was performed to obtain the residual stress field and quantify the influence of the geometrical parameters of the swage in the stress field. Gibson achieved that an increment of the inlet and outlet angles and a reduction of the length of the straight zone generated an increase of the residual hoop stresses at the bore of the hole. Moreover, these increments of the inlet and outlet angles and the reduction of the length of the straight zone generated a reduction of the yielded area after the cold expansion process.

In the case of the cold expansion of riveted holes in aeronautics, there are less research related to the influence of the mandrel geometrical parameters on the residual stresses. There are investigations using steel balls as mandrels [15] or swages, as previously shown in Figure 5 [16], but there is no systematic FEM analysis such as the Gibson’s study applied to small holes.

Starting from Gibson’s study on a parametric analysis of the mandrel geometry and its influence on in the residual stresses [13,14], this paper is focused on the specific cold-forming application for small holes drilled in thick plates. Similarities and differences in the cold-forming process applied to small holes and thick-walled cylinders were performed to verify or refute the applicability of the conclusions published by Gibson et al. for both scenarios.

## 2. Materials and Methods 

This investigation was centred on a parametric numerical analysis through a finite element method (FEM) of the mandrel geometrical data during a cold expansion process for a small hole. An ANSYS v19 software was used to simulate the cold-forming process. The evaluated mandrel geometrical parameters were: the inlet angle, the length of the straight zone and the interference between the swage and the hole. The outlet angle was fixed at *β* = 3°. A full factorial DOE (Design of Experiments) was designed to analyse every possible combination of the geometrical parameters (inlet angle, length of the straight zone and diametric interference).

Table 1 summarized the geometrical data included in the performed FEM analysis: four levels for the inlet angle (*α*), three levels for the straight zone length (*SZ*), and three levels for the diametric interference (*int*). A total number of 36 simulations for the cold expansion forming process were done (full factorial DOE). The selected ranges for each geometrical parameter were based on the optimum values deduced in previous investigations by Gibson [13] and O’Hara [17].

**Figure 6:**
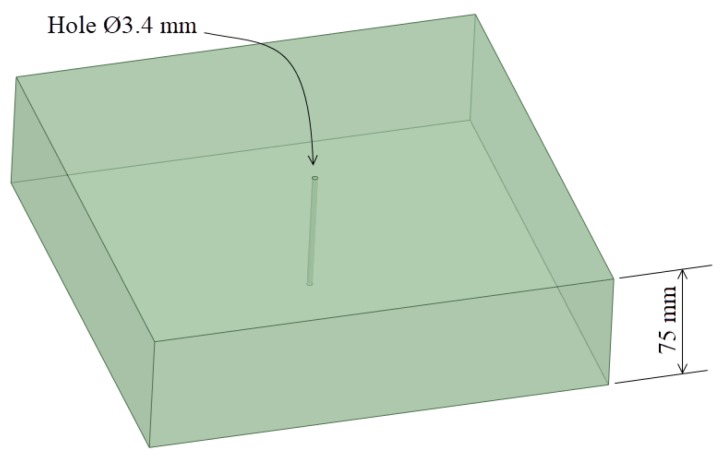
Sketch of the drilled plate.

For the plate geometry, a high ratio *L* = *t/D_hole_* = 22 was selected to consider a thick plate (where *t* = 75 mm was the plate thickness and *D_hole_* = 3.4 mm was the hole diameter; see Figure 6). As mentioned previously, the aim of the *L* ratio was to avoid the effect of the hole ends in order to evaluate and compare the results with the thick-walled cylinders.

In the specific case of the small holes, mandrels are usually manufactured with high strength steels, and considering the advances of powder metallurgy for tungsten carbide parts development, in this paper, a CTS12L alloy manufactured by Ceratizit [18] was selected for the high-strength and high-stiffness values (ultimate tensile strength of *σu* = 3500 MPa and Young’s modulus of *E* = 630 GPa).

Moreover, the material selected for the plate was the precipitation hardening stainless steel 15-5 PH H1025. Whose mechanical properties were listed in Table 2 as specified in the AMS5659 standard [19] As a note, it is important to point out that the alteration of plate material with lower plastic mechanical properties would derive in a reduction of the maximum residual stresses that cold expansion process could obtain, but the influence of each geometrical parameter evaluated in this investigation would have similar tendencies in the residual stresses.

The stress-strain curve of the considered steel was approximated with the Ramberg–Osgood hardening law [20] (see Equation (1)). Equation (2) deduced by Kamaya et al. [21] was used to calculate the hardening coefficient *n*, obtaining *n* = 30.3. The plastic hardening was simulated with a kinematic hardening model to introduce the Bauschinger effect in the cold forming process.

*ε* = *σ/E + ε_offset_ (σ/σ_y_*)*^n^* (1)

*n* = 3.93{*ln*(*σ_u_eng_/σ_y_*)}^−0.754^ (2)

where:

*E*: Young’s modulus,
*ε*: true strain,
*ε_offset_*: offset strain (0.002) to obtain the yield strength,
*σ*: true stress,
*n*: hardening coefficient,
*σ_u_eng_*: engineering ultimate tensile strength, and
*σ_y_*: yield strength.

**Figure 7:**
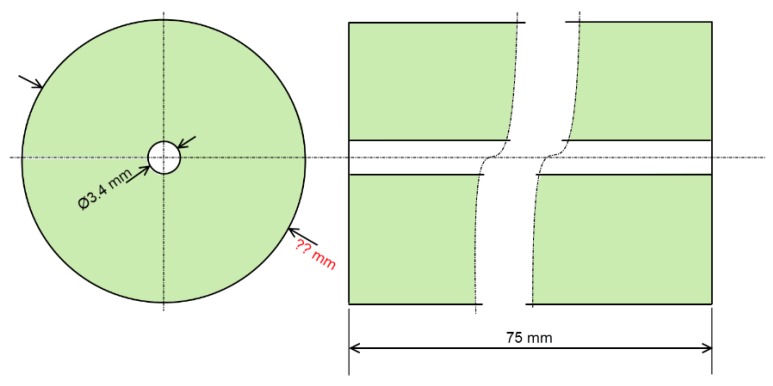
Geometry of the thick plate as an axisymmetric model.

To reduce the computational requirement of the numerical analyses, an axisymmetric model was considered. In the case of a thick plate, the outer diameter of the part needs to be defined (see Figure 7). The Lamé’s equation for the hoop stress on the inner diameter of a thick-walled cylinder with an inner pressure of 1000 MPa (see Equation (3)) was used to fix a specific value for the outer diameter.

*σ_hoop_* = (*D_ext_*^2^ + *D_int_*^2^)*/*(*D_ext_*^2^ − *D_int_*^2^)*·P_int_* (3)

where:

*D_ext_*: outer diameter,
*D_int_*: inner diameter,
*P_int_*: internal pressure, and
*σ_hoop_*: hoop stress.

**Figure 8:**
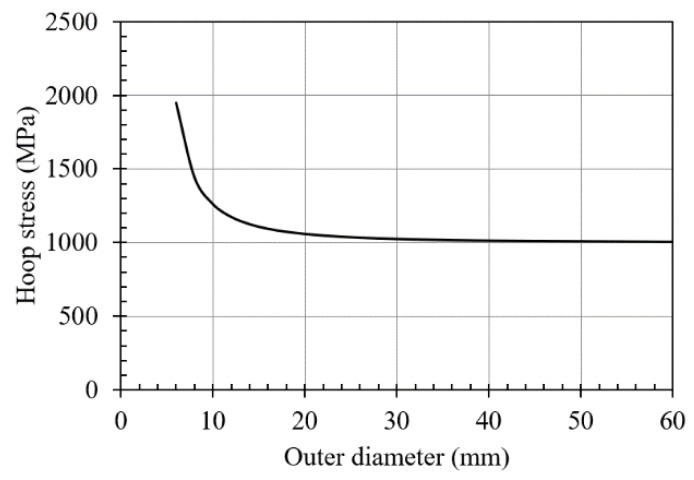
Hoop stress (*σ_hoop_*) in the inner diameter vs. the outer diameter.

**Figure 9:**
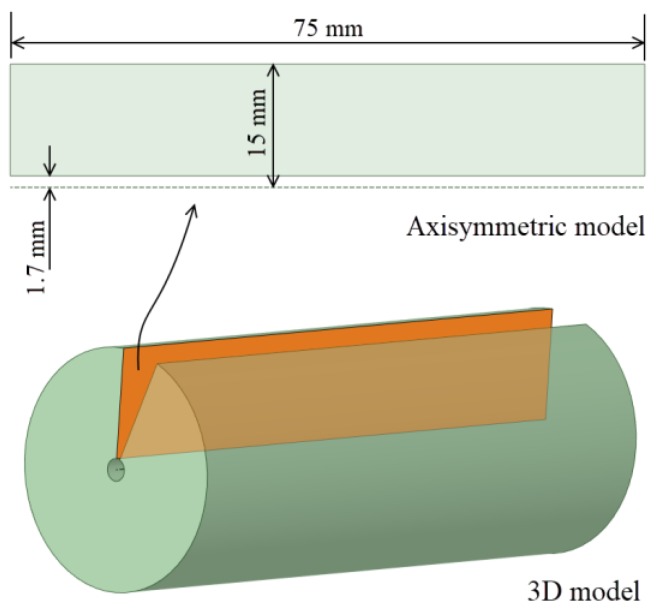
Geometry of the axisymmetric model.

Fixing the inner diameter at *D_int_* = 3.4 mm and the internal pressure at *P_int_* = 1000 MPa, the influence of the outer diameter (*D_ext_*) on the hoop stress at the hole’s bore tended to a stabilized value equal to the internal pressure (see Figure 8). Therefore, an outer diameter of 30 mm could be considered equivalent to a distant hole from any discontinuity (other holes or plate ends). Figure 9 shows the considered geometry for the axisymmetric model of the plate.

The FEM simulations were identified with the ID AeExxx-Ixxx-Lrxx, where: “Ae” represents the inlet angle of the mandrel followed by a numerical value in tenths of a degree; “I”, the diametric interference in hundredths of a millimeter; and “Lr”, the length of the straight zone in tenths of a millimeter. For example, the ID Ae200-I020-Lr15 matches with the mandrel of: inlet angle equal to *α* = 2°, diametric interference of *int* = 0.2 mm and length of the straight zone of *SZ* = 1.5 mm.

**Figure 10:**
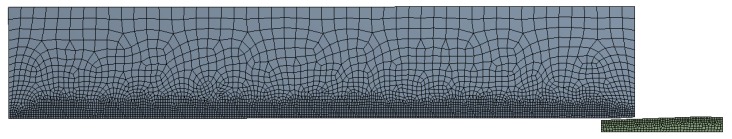
Mesh of the cold expansion simulation (*ms* = 0.2 mm).

**Figure 11:**
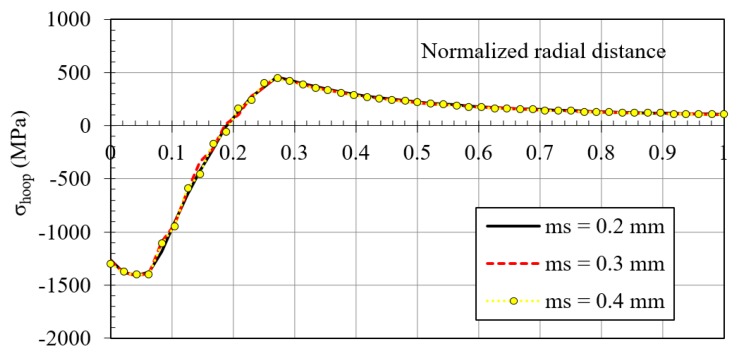
Sensitivity mesh analysis.

**Figure 12:**
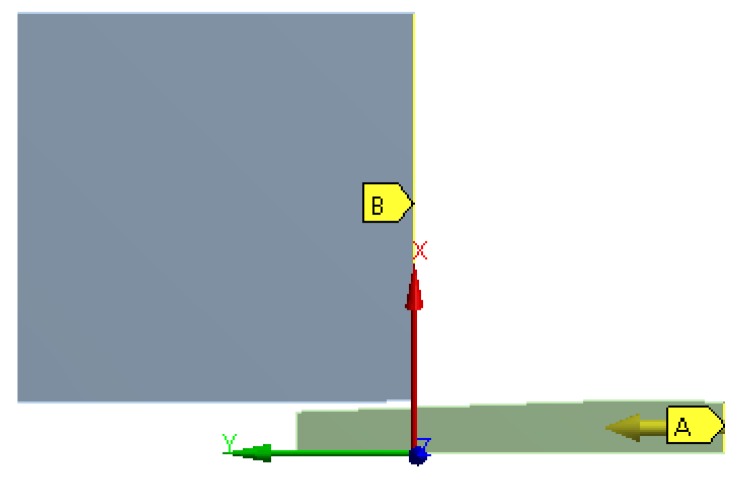
Boundary conditions of the FEM model.

For the contact between the mandrel and the hole, a friction coefficient *µ* = 0.05 (typical for a lubricated contact) was fixed for the simulations. A sensitivity mesh analysis was performed with fine mesh sizes of *ms* = 0.4, 0.3 and 0.2 mm established in the edge of the inner diameter of the plate and the outer face of the mandrel with Quad8 and Tria6 elements (see Figure 10). Figure 11 shows the residual hoop stresses obtained after the cold expansion process for the different *ms* values. These results showed no significant alterations and the mesh size of 0.2 mm was established as the standard for this FEM analysis, showing an orthogonal quality of *OQ* = {0.602, 1.000} and a skewness of *S* = {1.114 × 10^−3^, 0.591}. These mesh metrics are enough good to guarantee no convergence problems related with mesh quality. The boundary conditions of the model were a restricted longitudinal movement of the inlet edge of the plate (B location of Figure 12), and an imposed longitudinal movement of the outlet edge of the mandrel equal to 86 mm (A location of Figure 12).

**Figure 13:**
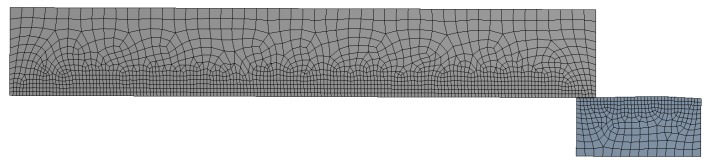
FEM model for the comparison with the Gibson FEM model.

**Figure 14:**
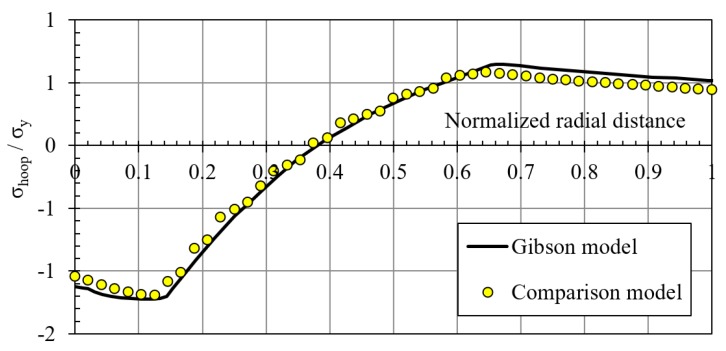
Comparison between residual hoop stresses obtained with the FEM model and the Gibson FEM model.

This FEM model was verified with a comparison with the Gibson FEM results [13], just changing the geometrical parameters and material properties of the previously shown model adapting it to the Gibson cylinder geometry. Table 3 shows the corresponding geometrical data and the mechanical properties and Figure 13 shows the FEM model used for this comparison. Figure 14 represents the comparison of the residual hoop stresses (normalized by the yield strength) versus the normalized radial position (from 0: inner radius to 1: outer radius). Similar results ensured the feasibility of the FEM model designed for this investigation.

## 3. Results

**Figure 15:**
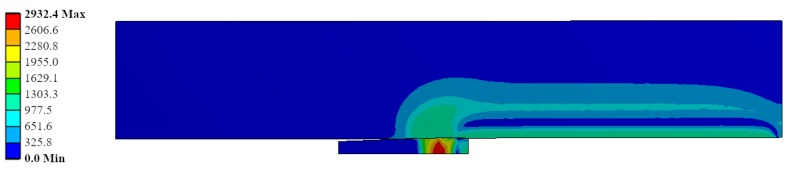
Von Mises stress (*σ_VM_*) during the cold expansion process.

**Figure 16:**
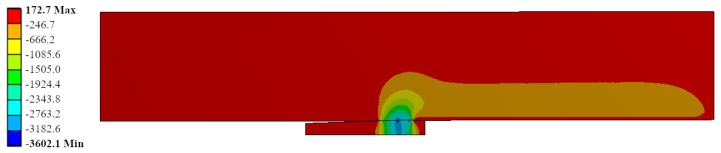
Radial stress (*σ_rad_*) during the cold expansion process.

**Figure 17:**
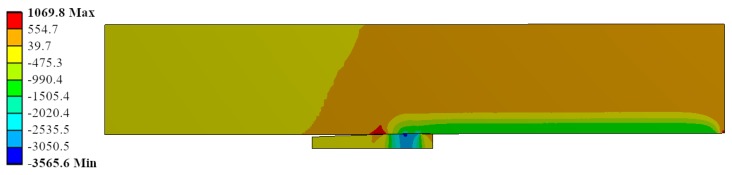
Hoop stress (*σ_hoop_*) during the cold expansion process.

**Figure 18:**
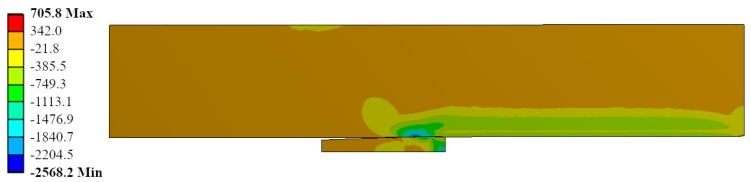
Longitudinal stress (*σ_long_*) during the cold expansion process.

**Figure 19:**
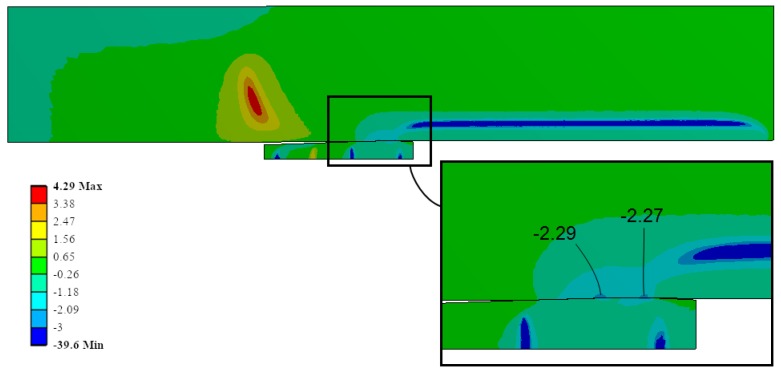
Triaxiality during the cold expansion process.

The results obtained from the FEM simulations of the mandrel geometrical data during the cold expansion process were reported in Figure 15, Figure 16, Figure 17, Figure 18 and Figure 19. Figure 15 shows the Von Mises stress during the cold expansion process simulation, where the mandrel has been pushed up to the half of the plate thickness. This figure represents the simulation ID Ae200-I020-Lr15 (inlet angle: 2.0°; diametric interference: 0.2 mm; and straight zone length: 1.5 mm). The most critical Von Mises stress is in the mandrel with values close to 3000 MPa. Therefore, this part needs to have a very high yield strength to guarantee no plastic behaviour of the mandrel during the process.

Radial stress field is represented for the same ID Ae200-I020-Lr15 in Figure 16. The most stressed location matched with the contact between the mandrel and the hole in the straight zone of the mandrel. The contact pressure reached up to −3600 MPa. This is feasible due to the high hydrostatic component in the stress field: the hoop stress reached −3600 MPa (see Figure 17) and the longitudinal stress showed values of −2600 MPa (see Figure 18).

Figure 19 shows the triaxiality for the same ID Ae200-I020-Lr15. Focusing the analysis in the region where the most critical stresses shown in the previous figures (i.e., the contact region between the mandrel and the hole), the triaxiality reached *FT* = −2.29 in the most critical location for the plate. This fact verified the previous hypothesis: with the high compressive hydrostatic component of the stress field (equivalent to a very negative triaxiality) was feasible to reach avery high contact pressure during the cold-forming process.

**Figure 20:**
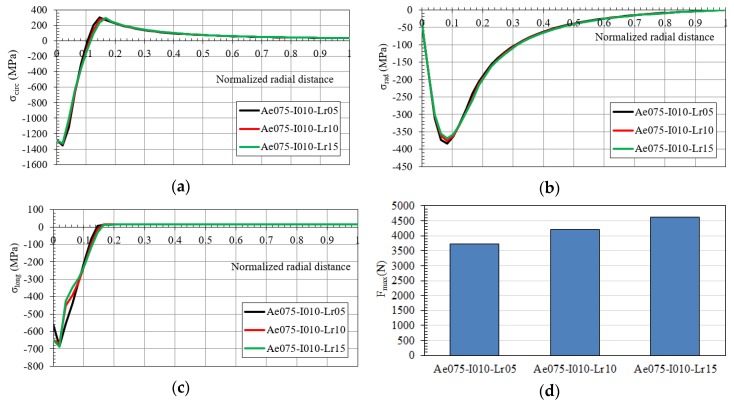
(**a**) Hoop; (**b**) radial; (**c**) longitudinal residual stresses along the normalized radial distance after the cold expansion process and (**d**) maximum pushing load in the mandrel (IDs Ae075-I010).

Figure 20a–c displays the residual hoop, radial and longitudinal stresses along the normalized radial distance in the plate (0, the hole radius; 1, the outer radius) after the cold expansion process for the IDs Ae075-I010-Lr05 /-Lr10 and /-Lr15. Changes in the length of the mandrel straight zone did not generate any significant variation in the residual stresses after the process. Figure 20d shows the maximum load needed to push the mandrel through the hole. This process parameter showed an increase with the increase of the length of the straight zone.

**Figure 21:**
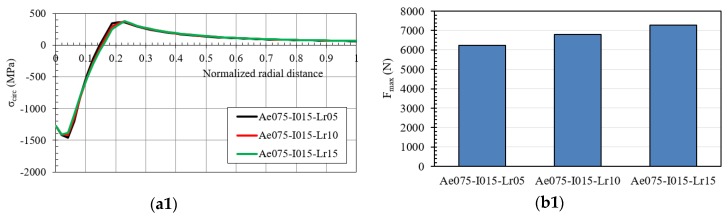

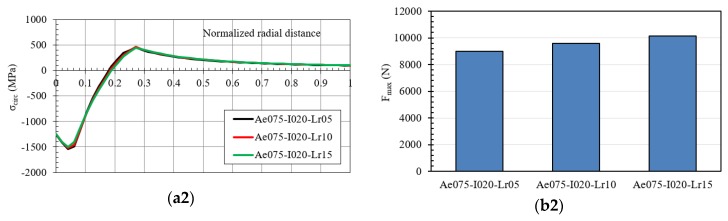
(**a**) Hoop residual stress along the normalized radial distance after the cold expansion process and (**b**) maximum pushing load in the mandrel [(**1**) IDs Ae075-I015; (**2**) IDs Ae075-I020].

Figure 21 shows the hoop residual stress and the maximum pushing load of the mandrel for the IDs Ae075-I015 and Ae075-I020. Similar results were obtained: the modification of the straight zone length did not generate any significant variation of the residual stresses on mandrels with an inlet angle of 0.75°, and diametric interferences of 0.15 and 0.20 mm with the hole. Interference growth generated:

1. An increase of the maximum compressive residual hoop stress: Ae075-I010: *σ_hoop_* = −1350 MPa, Ae075-I015: *σ_hoop_* = −1400 MPa and Ae075-I020: *σ_hoop_* = −1500 MPa.
2. An increase of the maximum pushing load of the mandrel.

**Figure 22:**
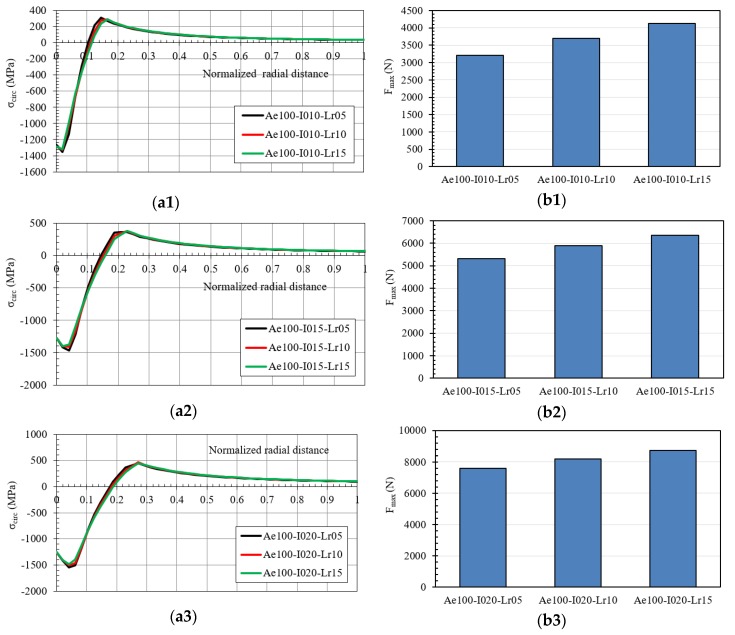
(**a**) Hoop residual stress along the normalized radial distance after the cold expansion process, and (**b**) maximum pushing load in the mandrel ((**1**) IDs Ae100-I010; (**2**) IDs Ae100-I015; (**3**) IDs Ae100-I020).

In Figure 22, the residual hoop stress and the maximum pushing load of the mandrel were reported for the IDs with an inlet angle of 1.0°: Ae100-I010, Ae100-I015 and Ae100-I020 with /-Lr05, /-Lr10 and /-Lr15. The variation of the straight zone length did not generate any change in the residual stresses, increasing only the maximum pushing load of the mandrel. Comparing these results with the inlet angle previously considered equal to 0.75°, the residual hoop stress did not change significantly, but the maximum pushing load of the mandrel diminished with the increase of the inlet angle.

**Figure 23:**
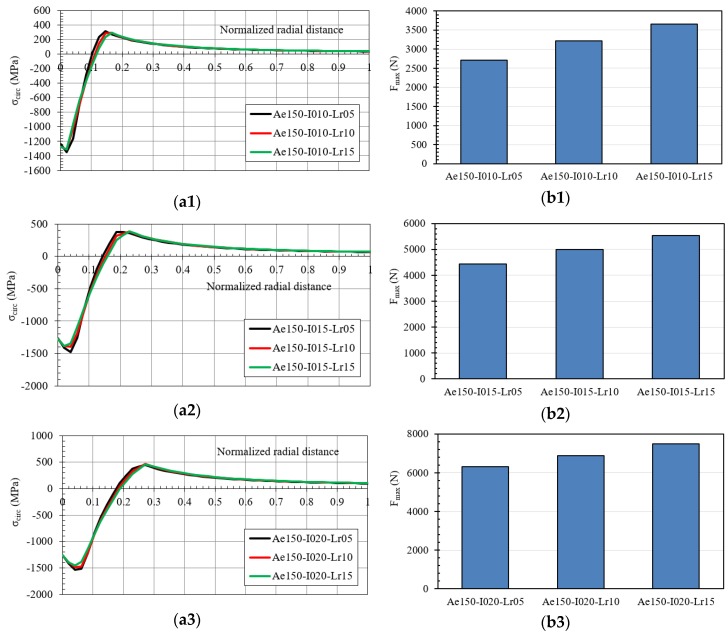
(**a**) Hoop residual stress along the normalized radial distance after the cold expansion process and (**b**) maximum pushing load in the mandrel ((**1**) IDs Ae150-I010; (**2**) IDs Ae150-I015; (**3**) IDs Ae150-I020).

**Figure 24:**
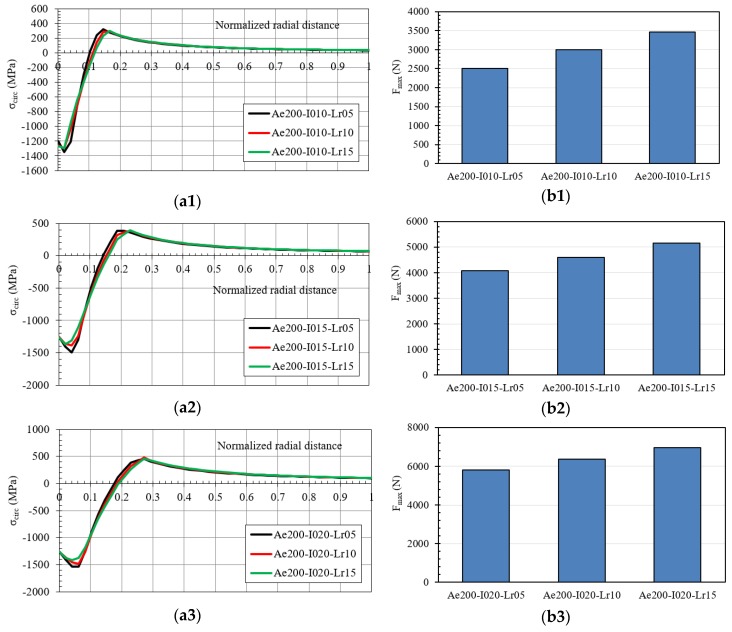
(**a**) Hoop residual stress along the normalized radial distance after the cold expansion process, and (**b**) maximum pushing load in the mandrel ((**1**) IDs Ae200-I010; (**2**) IDs Ae200-I015; (**3**) IDs Ae200-I020).

The results obtained for inlet angles equal to 1.5° and 2.0° are reported in Figure 23 and Figure 24, respectively.

1. Increase of the inlet angle generated a reduction of the maximum pushing load of the mandrel.
2. Increase of the straight zone length generated an increase of the maximum pushing load of the mandrel.
3. Variation of the inlet angle and the straight zone length did not show any significant influence in the residual stresses in the hole.

## 4. Discussion

**Figure 25:**
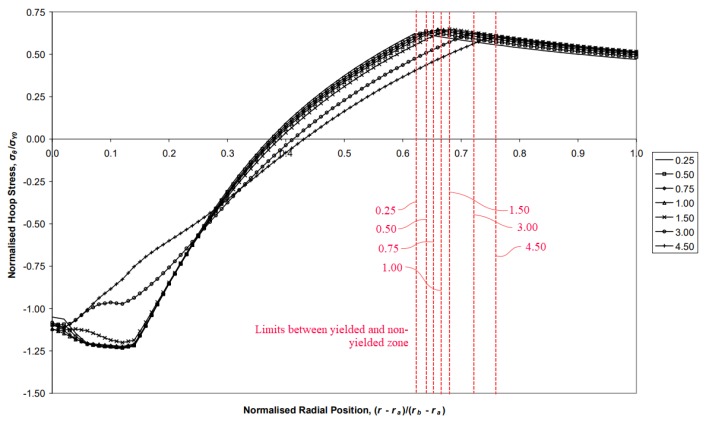
Normalized hoop residual stress along the normalized radial distance for different lengths of the straight zone in the Gibson et al. analysis [13].

The geometrical parameters of the mandrel evaluated in this study, inlet angle and the straight zone length, showed no significant influence in the residual stresses after the cold expansion process. This behaviour did not match the results showed by the numerical analysis performed by Gibson et al. [5,6] where there was significant variation of the hoop stress of thick-walled cylinders when the geometrical parameters of the swage were modified. Figure 25 shows the residual hoop stress obtained from the analysis performed by Gibson [5], where the influence of the straight zone length was analysed. An increase of this mandrel length did not significantly change the residual hoop stress, but from the length 1.0 up to 4.50, the compressive hoop stress at the smaller diameters of the cylinder showed an appreciable reduction. Therefore, the length value of 1.0 appeared as a limit.

This contradiction between the swaging of thick-walled cylinders and the cold expansion of small holes could have the explanation in the difference between the diameter ratio *K* of both geometries. The hole analysed in this investigation had a *K* = 30/3.4 = 8.8, whereas the Gibson’s study analysed a cylinder with *K* = 2.257. Other investigations, focused on the analysis of the geometrical parameters of the swage, followed similar *K* ratios: O’Hara (*K* = 2.257 [17]), Bihamta et al. (*K* = 3.18 [22]) and Alinezhad et al. (*K* = 3.18 [23]). Therefore, the *K* value used in thick-walled cylinders ranges from 2 to 4, a much smaller interval than the *K*’s value typically used in cold expansion processes. Could the *K* ratio be the origin of the previously mentioned contradiction? It could be answered with another question: what is the origin of the residual stresses after a swaging or cold expansion process? The remaining part of the cylinder or plate which has not been yielded during the process is the main part that is trying to return to its original position. However, the yielded zone does not want to return to that original position. Therefore, the non-yielded zone presses the yielded zone. If there is not a sufficient non-yielded zone, the residual compressive hoop stresses in the yielded zone will be significantly reduced. The vertical red dashed lines in Figure 25 shows the limit between the yielded and the non-yielded zones for the Gibson’s model. The increase of the straight zone length of the swage generates an increase of the yielded zone. Thus, the region of the cylinder which must press the yielded zone to generate the residual compressive hoop stress is smaller and smaller. When there is not a sufficient non-yielded zone, the residual compressive hoop stress ends diminish. This means that an increase of the straight zone length of the swage does not reduces the capability of the swage to yield the inner diameter of the hole. This capability is increased but if there is not sufficient outer diameter to press the hole after the cold-forming process, an increase of the yielded zone reduces the residual stresses. This could explain the lack of variation of the residual stresses of the small hole evaluated in this investigation. There is much more non-yielded zone in the plate, and the modification of the straight zone length of the mandrel did not reduce enough this zone to show consequences in the residual stresses.

In order to verify this approach, a FEM simulation of the cold expansion process evaluated in this investigation was repeated, but modifying the outer diameter from 30 mm to the obtained value with a *K* = 2.257 (the same *K* value used in the Gibson’s study): *D_ext_* = 3.4 × 2.257 = 7.67 mm. Two lengths of the straight zone were evaluated: 0.5 and 1.5 mm. The remaining geometrical parameters were:

- Diametric interference: 0.1 mm
- Inlet angle: 2°
- Outlet angle: 3°

**Figure 26:**
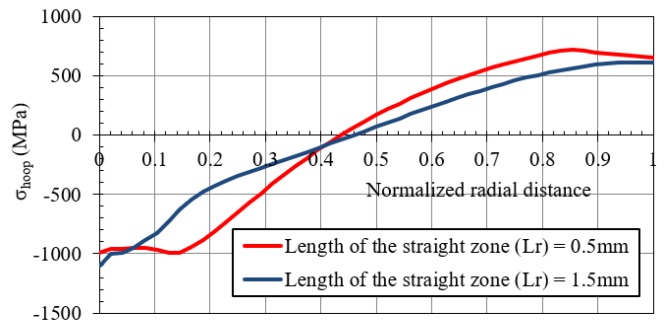
Hoop residual stress along the normalized radial distance after the cold expansion process with *K* = 2.257.

Figure 26 shows the residual hoop stress for straight zone lengths equal to 0.5 and 1.5 mm) along the normalized radial distance for the cold expansion process with a diameter ratio of *K* = 2.257 instead of *K* = 8.8 used in this investigation. The reduction of the compressive residual hoop stress for the inner diameters for *SZ* = 1.5 mm is similar to the behaviour shown by Gibson’s model, but the reason for this reduction is not justified only by the increase of the straight zone length. This increment generated an extension in the diameter of the yielded zone and the absence of enough non-yielded material to press the rest of the cylinder was the main cause for this reduction of the compressive residual hoop stresses. Consequently, the dependency of the residual hoop stresses with the straight zone length also depends on the *K* ratio.

In a cold expansion cold-forming process, the *K* ratio could show small values if the formed hole is close to any discontinuity (such as another hole or the end of the plate). It means that the influence of the straight zone length of the mandrel should be considered in those scenarios.

## 5. Conclusions

The main objective of this investigation was focused on a parametric analysis of the influence of the mandrel geometry in the residual stresses obtained after a cold expansion process of small holes with high ratio of plate thickness vs. hole diameter. Related to this general objective, this research has concluded the following:

1. *K* ratio has a considerable weight on the influence of the mandrel geometrical parameters for the residual stresses. High values of *K* ratio promote non-significant influence on the inlet angle variation and the straight zone length of the mandrel for the residual stresses of the cold-forming process.
2. Smaller inlet angles and higher the straight zone lengths generate an increase of the boundary diameter between the yielded and non-yielded zones of the plate. If *K* ratio is small enough, the non-yielded zone has not the capability to press the yielded zone and, consequently, the residual hoop stresses of the inner diameters diminishes.
3. The non-significant influence of the inlet angle and the straight zone length for the residual stresses of holes with high *K* ratios could mean that these values are not critical for the process, but the influence of these parameters on the pushing load of the mandrel is enough significant to consider them during any design. This numerical investigation showed that reducing the straight zone length and increasing the inlet angle, the pushing load is significantly reduced.

## Figures and Tables

**Table 1:** Mandrel geometry analysed in the parametric FEM simulations.

Geometrical Parameters	Value
Inlet angle (*α*)	0.75, 1.00, 1.50, 2.00°
Length of the straight zone (*SZ*)	0.5, 1.0, 1.5 mm
Diametric interference (*int*)	0.10, 0.15, 0.20 mm

**Table 2:** Mechanical properties of stainless steel 15-5PH H1025 (AMS5659).

Young’s modulus (GPa)	190
Yield strength (MPa)	1000
Ultimate tensile strength (MPa)	1069
Elongation at fracture (%)	12

**Table 3:** Geometry and mechanical properties of the Gibson FEM model [13].

**Cylinder**	
Young’s modulus (GPa)	209
Poisson’s ratio	0.3
Yield strength (MPa)	1195
Plastic modulus (GPa)	3.723
**Mandrel**	
Young’s modulus (GPa)	500
Poisson’s ratio	0.24
**Geometry**	
Cylinder inner radius (mm)	52.5
Cylinder outer radius (mm)	131.25
Cylinder length (mm)	525
Mandrel outer radius (mm)	53.81
Inlet angle (°)	1.5
Outlet angle (°)	3.0
Length of the straight zone(mm)	6.3
